# CUNR scoring system for the prediction of lateral lymph node metastasis in papillary thyroid carcinoma

**DOI:** 10.18632/oncotarget.22772

**Published:** 2017-11-30

**Authors:** Jianyong Lei, Gengpeng Li, Zhihui Li, RX Rong, Jingqiang Zhu

**Affiliations:** ^1^ Thyroid and Parathyroid Surgery Center, West China Hospital of Sichuan University, Chengdu 610041, China

**Keywords:** papillary, thyroid carcinoma, lateral, lymph node, metastasis

## Abstract

**Objective:**

Our present study aimed to evaluate and compare the number and rate of central lymph node metastases (LNMs) for the prediction of lateral LNM (LLNM) in papillary thyroid carcinoma (PTC) and to develop a scoring system.

**Results:**

Capsule invasion, tumor location in the upper portion of the thyroid, an ipsilateral central compartment LNM number ≥3, and an ipsilateral central compartment LNM rate of ≥56% were identified as significant independent predictors of ipsilateral lateral LNM in PTC. The predictive ability of an ipsilateral central compartment LNM rate ≥56% (area under the curve (AUC) = 0.802) was better than that of an ipsilateral central compartment LNM number ≥3 (AUC = 0.755). The ROC curves identified the best index point (CUNR) to distinguish the presence or absence of ipsilateral LLNM as 11, which has a high sensitivity (0.860) and a low false-negative rate (0.100, 1-Specificity). These findings were supported by the validation cohort.

**Conclusions:**

Patients with a CUNR index point equal to or greater than 11 and ipsilateral lateral lymph node dissection should be considered for a diagnosis of LLNM.

**Patients and Methods:**

A total of 1,281 PTC patients were included and divided into two groups: those with a presence of LLNM (*n* = 222) and those with an absence of LLNM (*n* = 1059). Univariate and multivariate analyses were performed to detect the risk factors for LLNM, and receiver operating characteristic (ROC) curves were used to detect the best cutoff values of these predictors. Additionally, a scoring system for the odds ratio (OR) of independent factors was developed and validated in an independent cohort of PTC patients (*n* = 560).

## INTRODUCTION

Papillary thyroid carcinoma (PTC) is the most common primary endocrine-related malignancy because it accounts for approximately 90% of all thyroid cancer cases [1]; currently, the incidence of PTC is rapidly increasing worldwide [2], particularly in Asian countries [3]. PTC is the most typical prolymphatic tumor because 20–90% regional lymph node metastases (LNM) are usually present at diagnosis [4, 5]. Additionally, accumulating evidence shows that lymph node metastases adversely affect survival, particularly in older patients with larger tumors and extrathyroidal extension [6]. Thus, complete resection of the primary tumor and metastatic lymph nodes is a prerequisite for subsequent thyroid-stimulating hormone (TSH) suppression and iodine 131 therapies. LNM is often elusive and difficult to detect and diagnose with preoperative ultrasonography, particularly in the lateral neck compartment, which exhibits variable sensitivities that range from 37 to 84% [6–8].

Prophylactic central lymph node dissection has been recommended to improve the prognosis of these patients. In general, PTC metastasis initially occurs in the central compartment (VI) and then spreads to the lateral compartment of the neck [9]; in rare cases, lateral lymph node metastasis (LLNM) skips over the central neck compartment (skip metastasis) [10, 11]. Previous studies have proven that the presence of central neck lymph node metastasis is valuable in the prediction of LLNM [9, 12–15]. Significant positive values for the ability of central lymph nodes to predict LLNM ranged from 2 to 5 [14, 16, 17]. However, until now, no study has reported the predictive significance of the central lymph node metastatic (LNM) ratio on LLNM, and no study has compared the number and efficiency of the central LNM ratio and number in the prediction of LLNM. The current study was designed to investigate the significance of the central LNM ratio in the prediction of LLNM and to compare the effectiveness of using the central metastatic lymph node number and ratio. In addition, we developed a scoring system based on the odds ratio (OR) of the risk factors to predict LLNM.

## RESULTS

### Patient demographics and tumor characteristics

The clinical and pathological characteristics of the 1,281 PTC patients enrolled in this study are shown in Table 1. In all, 343 (26.8%) patients were men, and 939 (73.3%) were women; the mean age was 40.5 years, and 439 (34.3%) patients were older than 45 years. All the patients underwent conventional open surgery, and Hashimoto’s thyroiditis (HT) was present in 336 (26.2%) patients with PTC. Chronic disease, including hypertension and diabetes, among others, was present in 216 (16.9%) patients with PTC. Most of the cases (859, 67.1%) showed unifocality, and 447 cases (34.9%) exhibited histological capsule invasion. Additionally, only 33 (2.6%) patients demonstrated distant metastases prior to surgery. The overall rate of LLNM was 222 among 1,281 patients (17.3%).

**Table 1:** Demographics and tumor characteristics of patients with PTC (*n* = 1281)

Characteristics	Results
Age at diagnosis (mean ± SD, years) Gender (M/F) Race (Han/others) BMI (kg/m^2^) Chronic disease (yes/no) Autoimmune thyroid disease (yes/no) Graves’ disease (yes/no) Nodular goiters (yes/no) NLR (mean ± SD) PLR (mean ± SD) TSH level (mU/L, mean ± SD) FT4 level (pmol/L, mean ± SD) FT3 level (pmol/L, mean ± SD) Multifocality (yes/no) Bilaterality (yes/no) Capsule invasion (yes/no) Extrathyroid extension (yes/no) Total tumor size (mean ± SD, mm) Largest tumor size (mean ± SD, mm) Primary tumor location (Upper/Middle/Lower) Tumor extension (T1/T2/T3/T4)	40.5 ± 13.6 342/939 1267/14 22.7 ± 3.6 216/1065 946/336 24/1257 600/681 1.9 ± 0.9 108.3 ± 51.0 3.2 ± 3.0 17.4 ± 5.6 4.9 ± 0.7 211/1070 115/1166 447/834 64/1217 14.9 ± 10.5 14.0 ± 9.8 522/397/362 448/114/539/180

Chronic disease: hypertension, diabetes and others;

SD: standard deviation; BMI: body mass index; NLR: neutrophil-to-lymphocyte ratio; PLR: platelet-to-lymphocyte ratio; TSH: thyroid stimulating hormone; FT3: free triiodothyronine; FT4: free thyroxine.

### Associations between the clinicopathological characteristics and LLNM in patients with PTC

**Figure 1:**
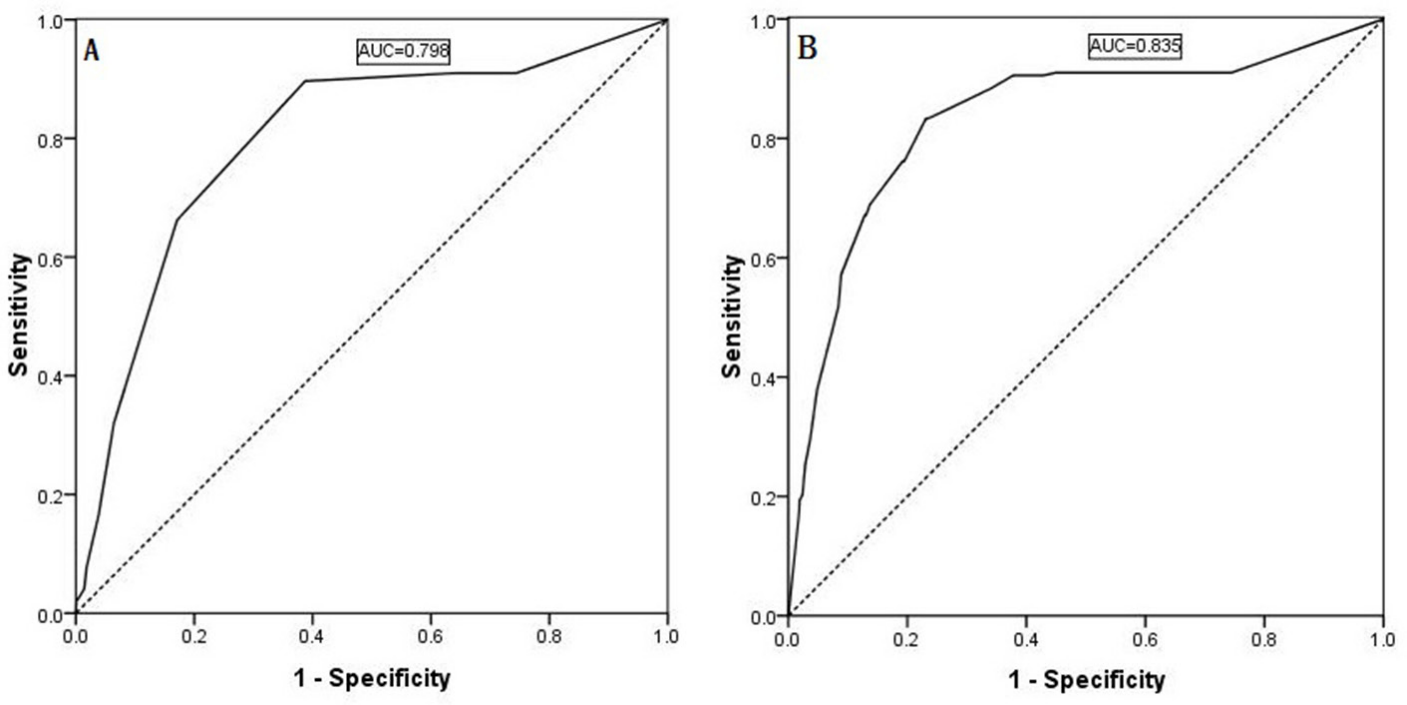
Predictability of ipsilateral lateral LNM shown by an ROC curve for the ipsilateral central compartment LNM number (**A**, AUC: 0.798) and the ipsilateral central compartment LNM rate (**B**, AUC: 0.835).

**Figure 2:**
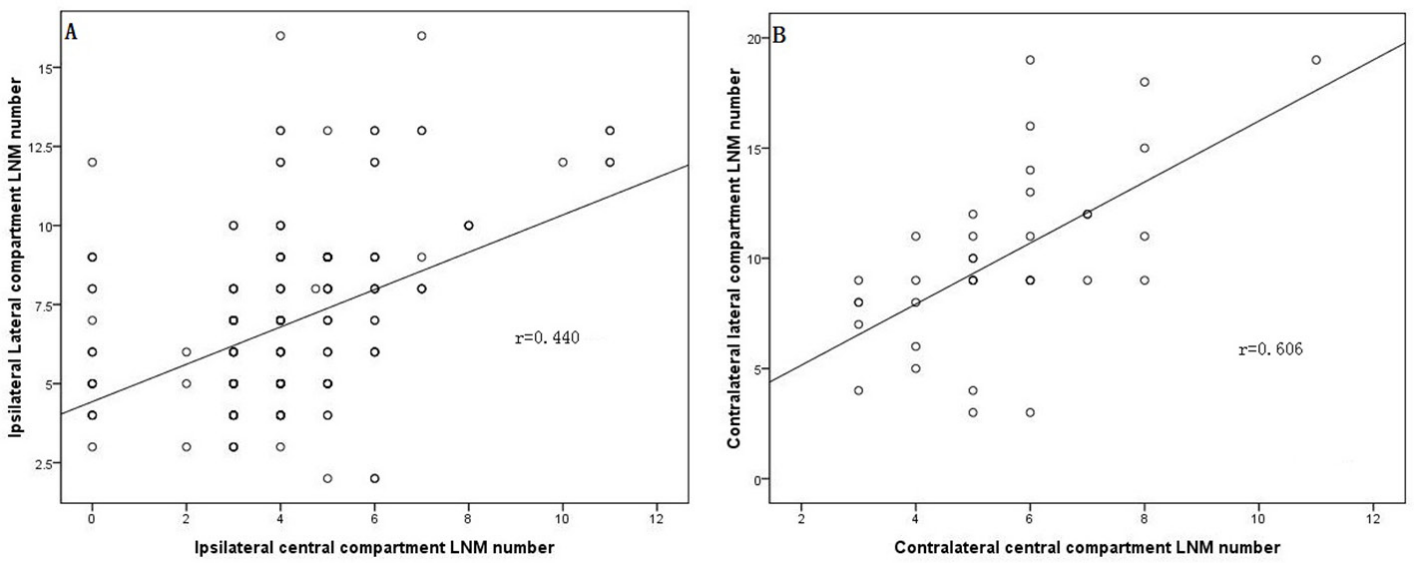
Ipsilateral and contralateral lateral compartment LNM numbers were linearly correlated with the ipsilateral (**A**, *r* = 0.440) and contralateral central compartment LNM numbers (**B**, *r* = 0.606).

Two hundred twenty-two patients (17.3%) were demonstrated to have LLNM in the final histopathological examination, whereas 1,050 patients (82.7%) demonstrated no evidence of LNM at least six months after surgery. As shown in Table 2, nodular goiters (*p =* 0.037) or Graves’s disease (*p =* 0.038), capsule invasion (*p <* 0.001), tumor location in the upper portion of the thyroid (*p =* 0.004), an ipsilateral central compartment LNM number ≥3 (*p <* 0.001), and an ipsilateral central compartment LNM rate ≥56% (*p <* 0.001) were significantly associated with a higher prevalence of LLNM in the univariate analysis. The cutoff values of the ipsilateral central compartment LNM number and rate were calculated from the ROC curve, as shown in Figure 1A and 1B. In the multivariate analysis shown in Table 3, capsule invasion (OR = 2.683, *p <* 0.001), tumor location in the upper portion of the thyroid (OR = 2.261, *p =* 0.002), an ipsilateral central compartment LNM number ≥3 (OR = 2.784, *p =* 0.003), and an ipsilateral central compartment LNM rate ≥56% (OR = 7.950, *p <* 0.001) were significant independent predictors of ipsilateral LLNM in PTC. Additionally, Pearson’s correlation analysis indicated that the ipsilateral and contralateral lateral compartment LNM numbers were linearly correlated with the ipsilateral and contralateral central compartment LNM numbers, respectively, as shown in Figure 2A and 2B (*r* = 0.440 and *r* = 0.606, all *p <* 0.001).

**Table 2:** Univariate analysis of LLNM in patients with PTC

Variable	Ipsilateral		*p* value
	Presence of LLNM (*n* = 222)	Absence of LLNM (*n* = 1059)	
Age (≤45/>45 years) Sex (male/female) Race (Han/other) BMI (<24/≥24kg/m^2^) Chronic disease (yes/no) HD (yes/no) Graves’ disease (yes/no) Nodular goiters (yes/no) NLR (≤2/>2) PLR (≤200/>200) TSH level (≤4.2/>4.2 mU/L) Multifocality (yes/no) Capsule invasion (yes/no) Extrathyroid extension (yes/no) Total tumor size (≤10 mm, >10 mm) Primary tumor size (≤10 mm, >10 mm) Tumor extension (T1–T2/T3–T4) Primary tumor location (upper/middle or lower) Ipsilateral central compartment LNM number (<3/≥3) Ipsilateral central compartment LNM rate (<56%/≥56%) Contralateral central compartment LNM (<3/≥3) Contralateral central compartment LNM rate (<69%/≥69%)	150/72 59/163 221/1 149/73 29/193 164/58 8/214 118/104 136/86 215/7 186/36 37/185 136/86 6/216 87/135 99/123 87/135 113/109 23/199 37/186 113/109 158/64	692/367 282/777 1046/13 714/345 187/872 782/277 16/1043 482/577 694/365 1007/52 848/211 885/174 311/748 58/1001 487/572 523/364 475/584 413/646 649/410 811/248 798/261 970/89	0.526 0.987 0.312 0.930 0.097 0.836 0.037^*^ 0.038^*^ 0.226 0.256 0.252 0.931 <0.001^*^ 0.085 0.064 0.177 0.122 0.004^*^ <0.001^*^ <0.001^*^ <0.001^*^ <0.001^*^

LLNM: lateral lymph node metastasis; BMI: body mass index; HD: Hashimoto thyroiditis; NLR: neutrophil-to-lymphocyte ratio; PLR: platelet-to-lymphocyte ratio; TSH: thyroid stimulating hormone; LNM: lymph node metastasis

**Table 3:** Multivariate analyses of factors contributing to LLNM in PTC

Variables	Odds ratio		95% CI	*p* value
**Ipsilateral lateral compartment LNM risk factors**				
Capsule invasion (yes/no)		2.683	1.895–3.798	<0.001^*^
Primary Tumor location (upper/middle or lower)		2.261	1.348–3.792	0.002^*^
Ipsilateral central compartment LNM number (≥3/<3)		2.784	1.418–5.464	0.003^*^
Ipsilateral central compartment LNM rate (≥56%/<56%)		7.950	4.495–14.061	<0.001^*^
**Contralateral lateral compartment LNM risk factors**				
Contralateral central compartment LNM number (≥3/<3)		9.865	7.564–9.992	<0.001^*^
Contralateral central compartment LNM rate (≥69%/<69%)		64.068	8.667–473.584	<0.001^*^

LNM: lymph node metastasis; LLNM: lateral lymph node metastasis.

Univariate and multivariate analyses were performed to examine the risk factors for contralateral lateral compartment LNM and revealed that a contralateral central compartment LNM ≥3 (OR = 9.865, 95% CI, 7.564–9.992, *p <* 0.001) and a contralateral central compartment LNM rate ≥69% (OR = 64.068, 95% CI, 8.667–473.584, *p <* 0.001) were significant predictors of contralateral LNM.

### ROC curve

**Figure 3:**
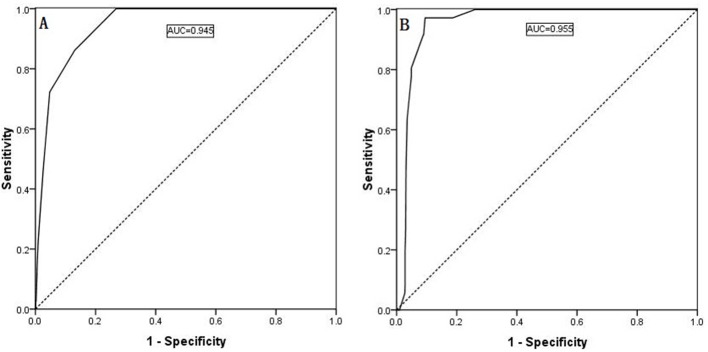
Predictability of contralateral lateral LNM shown by an ROC curve for the contralateral central compartment LNM number (**A**, AUC: 0.945) and the ipsilateral central compartment LNM rate (**B**, AUC: 0.955).

ROC curves were generated, and area under the curve (AUC) calculations were performed for the regression models of the continuous variables, as shown in Figure 1A and 1B. The Youden index was used to estimate the appropriate critical point. The ROC curve analysis identified 2.5 as the appropriate critical point for the ipsilateral central LNM number, and the ipsilateral central LNM rate was 0.560. Therefore, we defined the ipsilateral central LNM high number as ≥3, and the ipsilateral central LNM high rate as ≥56%. Based on the ROC curve analysis, 2.5 is the appropriate critical point for the contralateral central LNM number, and the contralateral center LNM rate was 0.690. Thus, we defined the contralateral central LNM high number as ≥3 and the contralateral central LNM high rate as ≥69%, as shown in Figure 3A and 3B.

**Figure 4:**
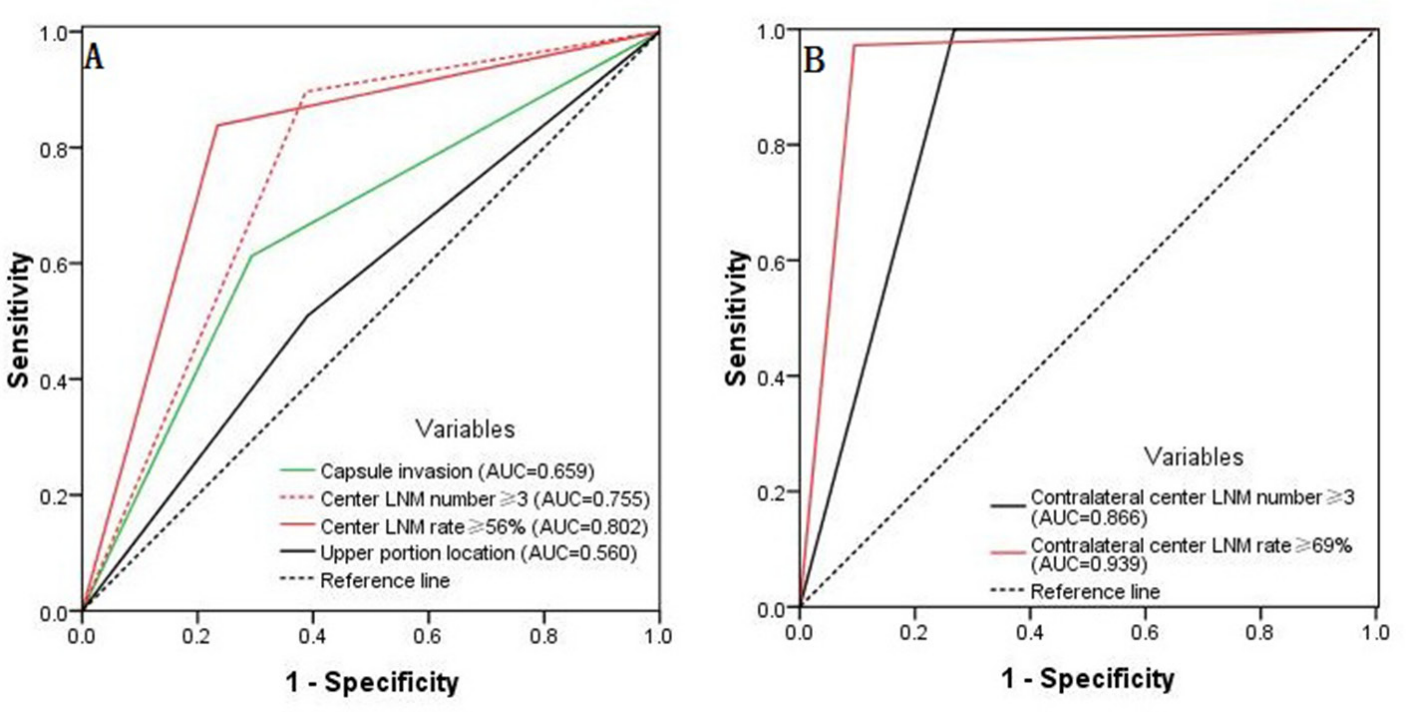
(**A**) The predictive factors for ipsilateral lateral compartment LNM were an ipsilateral central LNM rate ≥56%, an ipsilateral central LNM number ≥3, capsule invasion, and a tumor location in the upper portion of the thyroid. (**B**) The predictive factors for contralateral lateral LNM were a contralateral central LNM rate ≥69% and a contralateral central LNM number ≥3.

As shown in Figure 4A, a comparison of the four predictive factors for ipsilateral lateral compartment LNM revealed the following predictive factors: ipsilateral central LNM rate ≥56% (AUC = 0.802), an ipsilateral central LNM number ≥3 (AUC = 0.755), capsule invasion (AUC = 0.659), and a tumor location in the upper portion of the thyroid (AUC = 0.560). Moreover, the predictive factors of contralateral lateral LNM were a contralateral central LNM rate ≥69% (AUC = 0.939) and a contralateral central LNM number ≥3 (AUC = 0.866), as shown in Figure 4B.

The characteristics of ipsilateral lateral compartment LNM are shown in Table 4; single-level LNM was observed in 35 cases. The most common levels were Level IV (71.2%) and III (70.3%), followed by Level II (41.0%) and Level V (33.3%). Double-level (51.8%) LLNM was the most common type, followed by triple-level (26.1%), single-level (15.8%) and quadruple-level (6.3%) LLNM.

**Table 4:** Distribution of LLNM in 222 patients

Distribution (II–V)	Number of patients
Single level (II/III/IV) Lateral neck LN metastasis cases Level II Level III Level IV Level V Lateral compartment single-level metastasis Lateral compartment double-level metastasis Lateral compartment triple-level metastasis Lateral compartment four-level metastasis	10/4/17/4 91 156 158 74 35 115 58 14

### Index points for the distinction of the presence or absence of LNM

**Figure 5:**
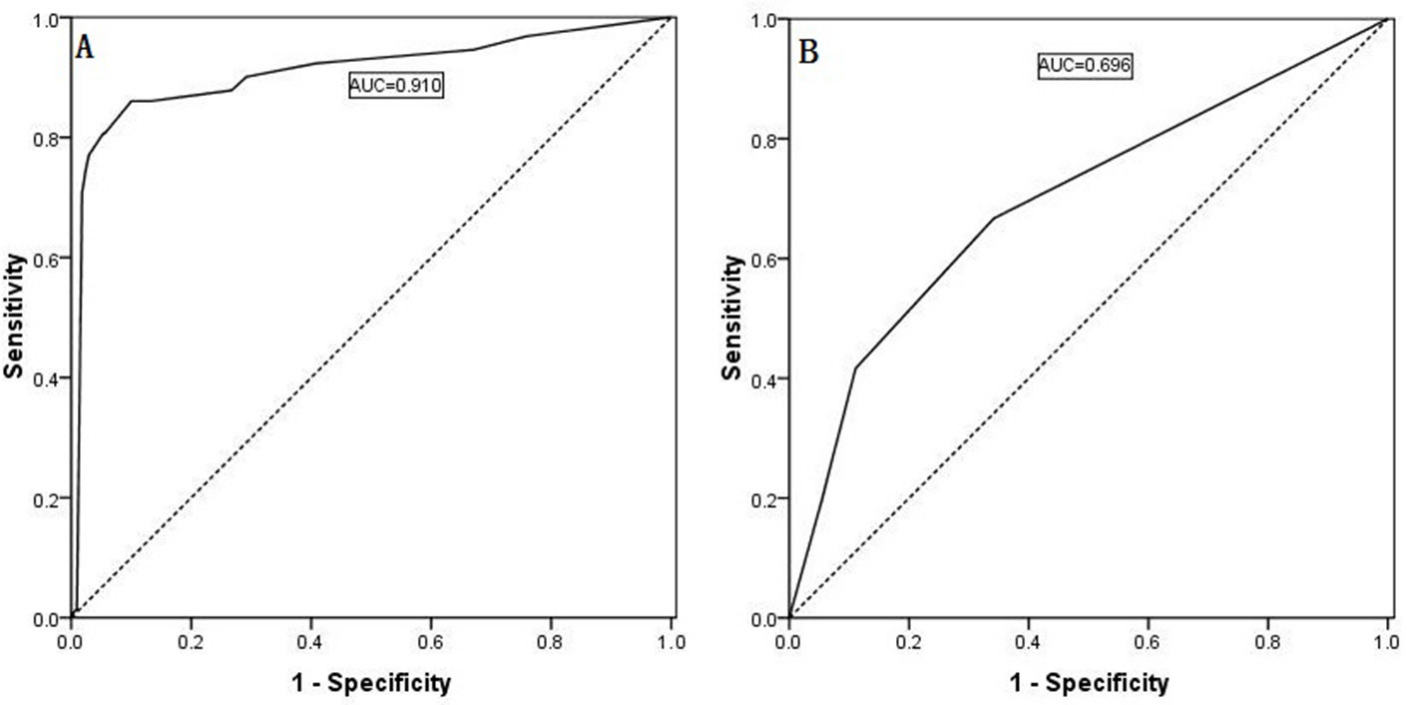
(**A**) Predictability of ipsilateral lateral LNM as shown by an ROC curve based on the scoring system. The best point with the highest sensitivity and the lowest false-negative rate was 11; the AUC was 0.910. (**B**) The calculated index points for contralateral lateral compartment LNM were 0, 10, 64, and 74.

According to the multiple logistic regression analysis, four risk factors and two risk factors were statistically significantly associated with ipsilateral lateral compartment LNM and contralateral lateral compartment LNM, respectively, and the estimate and cutoff points for each characteristic are shown in Table 5. The sum of the points (CUNR) was evaluated for its ability to distinguish between the presence and absence of lateral LNM. As determined using the ROC curves, the best point with high sensitivity (0.860) and a low false-negative rate (0.100, 1-Specificity) was 11, and the AUC was 0.910, as shown in Figure 5A. However, the calculated index points for contralateral lateral compartment LNM were 0, 10, 64, and 74, and the ROC curve is shown in Figure 5B; however, the calculated AUC is only 0.696, which is markedly lower than that for a contralateral central compartment LNM number ≥3 (AUC = 0.866) and that for a contralateral central compartment LNM rate ≥69% (AUC = 0.939).

**Table 5:** CUNR Index points for ipsilateral LLNM

Variables	CUNR	Odds ratio	Index points
**Ipsilateral lateral compartment LNM risk factors** Capsule invasion (yes/no) Primary tumor location(upper/middle or lower) Ipsilateral central compartment LNM number (≥3/<3) Ipsilateral central compartment LNM rate (≥56%/<56%) **Contralateral lateral compartment LNM risk factors** Contralateral central compartment LNM (≥3/<3) Contralateral central compartment LNM rate (≥69%/<69%)	C U N R	2.683 2.261 2.784 7.950 9.865 64.068	16/0 3/0 2/0 3/0 8/0 74/0 10/0 64/0

### Independent validation of our scoring system

**Figure 7:**
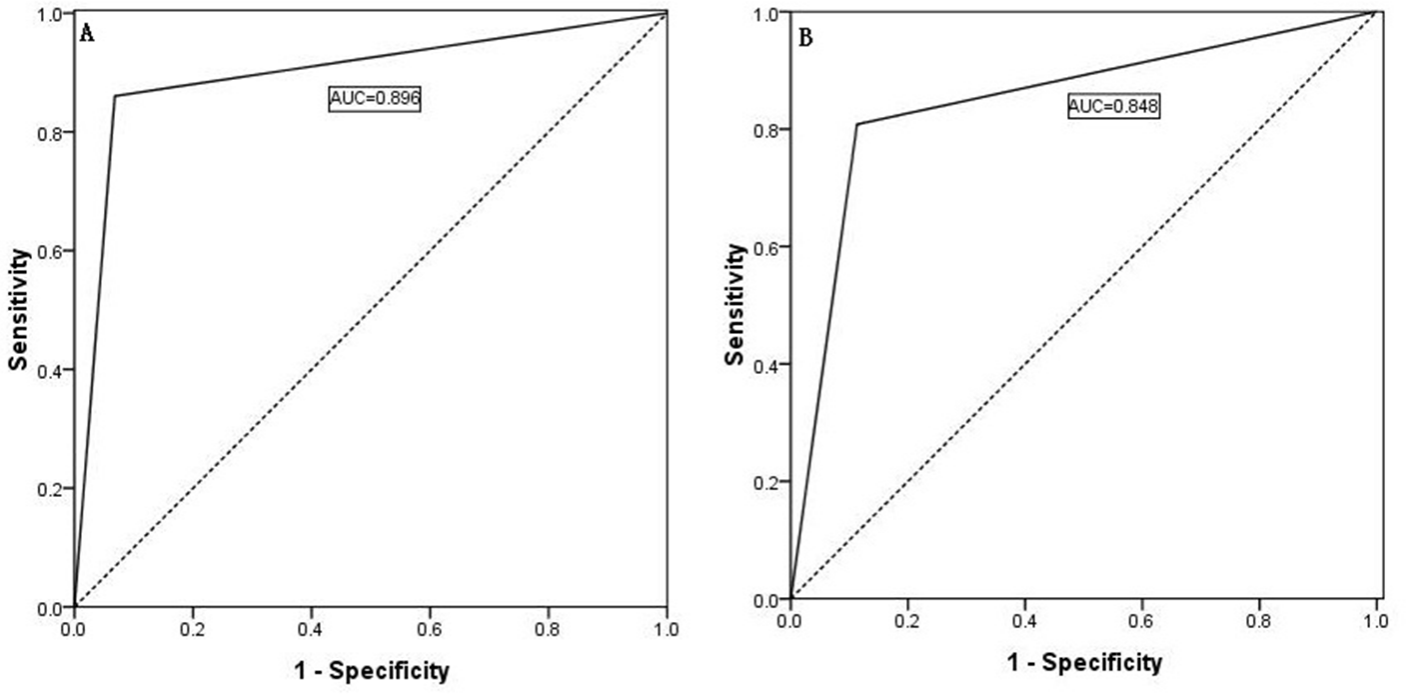
(**A**) Predictivity of the CUNR score index for ipsilateral lateral compartment LNM in the training cohort; (**B**) Predictivity of the CUNR score index in the validation cohort.

Our scoring system for the prediction of lateral LNM was validated by an independent cohort of PTC patients at our center (*n* = 560), and the results revealed that our scoring system performed equally well. The AUC of the CUNR scoring for the prediction of ipsilateral lateral LNM was 0.848 in the validated cohort (as shown in Figure 7B), and this value was comparable with that obtained with the training cohort (as shown in Figure 7A, AUC = 0.896; *p* value > 0.10).

### Long-term tumor-free survival

**Figure 6:**
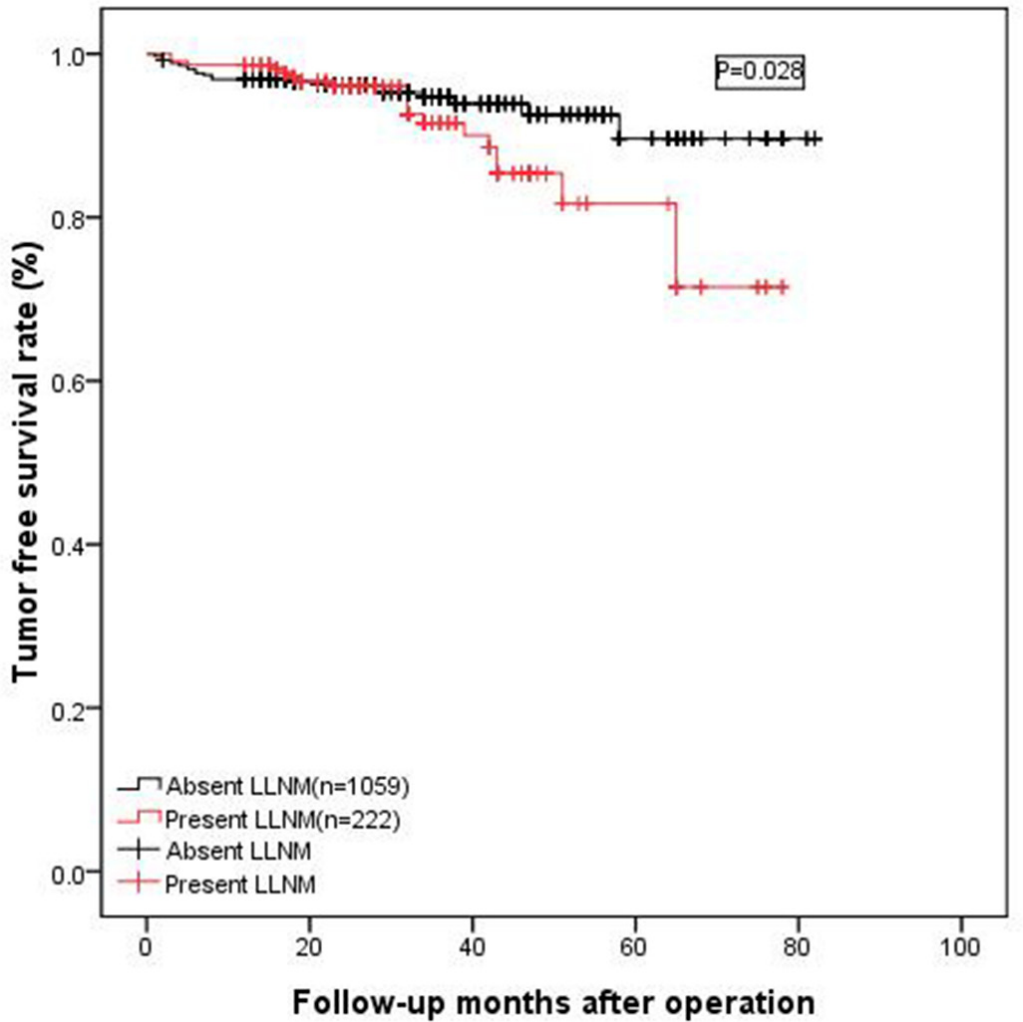
The tumor-free survival rate of patients without preoperative LLNM was significantly lower than that of patients with preoperative lateral LNM (*p* = 0.028)

Eight patients with distant metastasis were excluded from this analysis, 20 patients (9.0%) with preoperative LLNM were diagnosed with tumor recurrence or metastasis, and 57 (5.5%) patients did not present with preoperative LLNM at least six months before surgery. Additionally, the tumor-free survival in the group without preoperative LLNM was significantly less than that of the group with preoperative lateral LNM (as shown in Figure 6, *p* = 0.028). For the 77 patients with recurrence or metastasis, the most common site was the cervical lymph nodes, followed by the lung, bone and liver.

## DISCUSSION

PTC is one type of thyroid carcinoma that originates from follicular cells and that has a favorable long-term outcome. However, PTC frequently metastasizes to the cervical lymph nodes, and LNM is one of the significant predictive factors of a poor outcome, with high rates of recurrence, distant metastasis and the requirement for more aggressive management when LNM is suspected [18]; thus, we focused on LNM of PTC. Unfortunately, the status of LNM is not obvious, and the way in which the status is determined is inadequate. Due to its high specificity and positive predictive value, ultrasonography is an important tool for the detection of metastatic nodes [19]; however, ultrasonography has limitations, including variable sensitivities (37–84%) and difficulty in evaluating LNM [7, 8]. As shown in recent studies, routine unilateral central lymph node dissection does not increase the risk of complications but might help determine the central lymph node status and could provide more accurate cancer staging information [20, 21]. Thus, prophylactic central, but not prophylactic lateral, LND has been recommended for patients with PTC, and other clinicopathological predictive factors of LLNM might improve the selection of patients for lateral neck dissection.

Our hypothesis was that the number or rate of positive central lymph nodes is valuable in the prediction of the presence of LLNM. In general, cervical lymph node metastasis initially occurs in the central compartment lymph nodes and subsequently in the lateral neck compartment [9], whereas “skip metastasis” occurs in very few patients with PTC [22, 23]. Although this study is not the first to evaluate the ability of the number or rate of central LNM to predict LLNM [6, 15], to our knowledge, our study is the first to compare the ability of both the number and rate of central LNM for the prediction of LLNM; most importantly, we established index points based on the risk factors to distinguish patients with or without LNM. Moreover, we validated our CUNR scoring index independently in a large number of PTC patients. Our CUNR scoring system was valuable and feasible: first, unilateral central LND is routinely performed; and second, the frozen sections of the specimens dissected from the central compartment are analyzed in less than 30 minutes to determine the number and rate of central LNM [24].

As shown in previous studies, central lymph node metastasis is an important independent factor for LLNM, and most of these studies assessed the ability of the number of central LNMs to predict LLNM; however, the cutoff values vary. Machens *et al* [16] reported that the rates of LLNM increased from 45–69% to 100% in the ipsilateral neck and from 0–33% to 60–71% in the contralateral neck when patients had more than five central lymph nodes that were positive for metastases. Zeng *et al.* [14] and Xiao *et al* [17] reported that the presence of at least two positive central lymph nodes was an independent predictive factor for LLNM and should be valuable in the prediction of LLNM. Lee *et al.* [18] indicated that further treatment of the lateral compartment should be considered if the number of positive central lymph nodes is greater than two (≥3), which is a finding that was consistent with our results. However, our cutoff value demonstrated greater predictive ability. First, our total number of patients with PTC was larger than that in previous studies; second, the cutoff value was calculated from the ROC curve and was verified through univariate and multivariate analyses. Finally, the combination of all the predictive factors was used to establish a scoring system to predict ipsilateral LLNM.

Only a few studies have evaluated the central metastatic lymph node ratio. Zhu *et al.* [15] found that the high number for central LNM was ≥4 and that the high rate for central LNM was ≥40% for the prediction of LLNM. In our study, the cutoff value for the central LNM rate for the prediction of ipsilateral LLNM was 56%, which is higher than the value obtained in the study by Zhu *et al.* Moreover, univariate and multivariate analyses indicated that the number and rate of contralateral central compartment LNM were also risk factors for contralateral lateral compartment LNM; the OR of the rate of contralateral central compartment LNM was 64.068, which is significantly higher than the OR of the number of contralateral central compartment LNM (9.865). Thus, compared with the number of central LNM, the rate of central LNM was more valuable for the prediction of LLNM, including ipsilateral and contralateral LLNM.

The association between HT and LNM remains controversial. Patients with HT may have less extrathyroidal extension, less extranodal spread, a lower rate of central and lateral LNM, a lower TNM score [15], better prognosis, and a lower recurrence rate [25], which has led to the hypothesis that the lymphocytic response limits tumor growth and metastases [26]. However, in patients with PTC, LLNM and HT were more frequently observed in another study [27]. Capsule invasion proved to be an independent factor for the presence of LNM. In our present study, the univariate and multivariate analyses indicated that an ipsilateral central compartment LNM rate ≥56% (OR = 7.950), an ipsilateral central compartment LNM number ≥3 (OR = 2.784), capsule invasion (OR = 2.683) and a location of the primary tumor in the upper thyroid (OR = 2.261) were independent predictive factors of LLNM. Furthermore, an ipsilateral central compartment LNM rate ≥56%, which had the highest OR value, was a key factor in predicting ipsilateral lateral LNM. Based on the OR value, we established a scoring system and determined that the best cutoff value for the prediction of LLNM with high sensitivity (0.860) and specificity (0.900) was 11 (AUC = 0.910).

This study had several limitations, such as the inevitable inherent features of a retrospective and nonrandomized cohort study. Because LNM was evaluated only for therapeutic purposes at our center, residual subclinical LLNM might have been present; however, prophylactic LNM is not generally recommended by the ATA or ETA guidelines, and thus, this limitation is inevitable. Moreover, our predictive index point cannot replace ultrasonography, FNA or thyroglobulin (Tg) levels in the diagnosis of lateral LNM but might still be helpful. Therefore, larger randomized multicenter studies are needed to confirm our results.

The CUNR scoring system for the risk factors that predict LLNM might be another approach that can be used for the diagnosis of LLNM and for the guidance of lateral LND. Patients with index points ≥11 should be considered ipsilateral LLNM-positive; when the rate of contralateral central compartment LNM is ≥69%, contralateral LLNM should be considered. However, the CUNR scoring system should be used only as an adjunct to ultrasonography and pathology.

## PATIENTS AND METHODS

The institutional review board of West China Hospital approved this retrospective observational study, and the patients provided approval and written informed consent prior to surgery; further patient approval or informed consent was not required for our review of the patients’ medical records. From December 2008 to December 2015, 3,385 consecutive patients who underwent surgery for PTC at our center were included in the present study. The inclusion criteria for the present study were as follows: patients with PTC who underwent total or near-total thyroidectomy and patients who underwent bi-central lymph node dissection (LND). The exclusion criteria were as follows: patients with other types of thyroid malignancies, such as follicular or medullary carcinoma; patients with a history or file record of thyroid or neck surgery for nonthyroid head and neck carcinomas; and patients with bilateral lobe PTC. Based on these criteria, 1,281 patients with PTC were included in the present study.

Ultrasonography and neck-enhanced computed tomography (CT) or magnetic resonance imaging (MRI) were routinely performed on all patients prior to surgery, and the imaging results were co-evaluated by a radiologist and a surgeon with at least 10 years of experience with overall subject evaluation. In cases where PTC was suspected (presence of central necrosis or cystic changes, dense cortical enhancement, nonparallel shape, hypoechogenicity, irregular margins, or microcalcification), fine-needle aspiration biopsy (FNAB) and cytological BRAF^V600E^ mutation analyses were performed to confirm the diagnosis. All lateral neck lymph node compartment dissections were performed in patients with PTC who were confirmed to have clinical evidence of positive lateral neck nodes (no prophylactic lateral lymph node dissection) via FNAB or FNA thyroglobulin assessment; in other cases, an intraoperative frozen biopsy demonstrated the presence of LLNM. Level II, III, IV, and V LLNM cases were routinely dissected. Prophylactic bilateral central neck dissection (bi-CND) was performed on all of the patients with PTC and proven LLNM at our center, and prophylactic contralateral central compartment LN dissection was performed if prelaryngeal or pretracheal lymph node (LN) metastasis was diagnosed through the assessment of intraoperative frozen sections [28]. The central lymph node compartment was defined as the region superior to the thyroid cartilage notch, lateral to the carotid sheaths, posterior to the prevertebral fascia, including VI A (superficial right recurrent laryngeal nerve) and VI B (posterior of the right recurrent laryngeal nerve), inferior to the innominate vein, and inboard to the tracheal sidewall. All surgical procedures were performed by one of three experienced surgeons with at least 15 years of thyroid surgery experience. The histopathological evaluation of the thyroid specimens and lymph node metastases was performed independently by two experienced pathologists with at least 10 years of experience in the diagnosis of over 500 cases of PTC. We defined capsule invasion as invasion of the thyroid capsule without organ or soft tissue invasion, as observed on microscopy. Moreover, we defined extrathyroid extension as the presence of soft tissue/organ invasion, as shown on microscopy [29].

Postsurgical radioiodine ablation was applied according to the postoperative pathological findings, and postoperative TSH suppression therapy was based on the ATA guidelines [30]. We defined the central compartment as the region located cranially to both superior thyroid arteries and to the pyramidal lobe, caudally to the innominate vein, laterally to the carotid sheaths and dorsally to the prevertebral fascia [31]. We distinguished recurrence by the presence of residual tumor at a follow-up duration of more than six months after initial surgery [32].

To avoid overoptimistic results due to model development and evaluation using the same dataset, the predictive performance of the scoring system was assessed in an independent validation cohort.

All data were managed and analyzed using SPSS software (version 17; SPSS, Inc., Chicago, IL, USA). Continuous and categorical data are expressed as the means ± SD and rates, respectively, and the differences were compared and analyzed using the Mann-Whitney *U*-test and chi-squared test or Fisher’s exact test (two-tailed), if necessary. Univariate analyses were used to test the association with LLNM. Multivariate logistic regression was performed on all variables that were significant in the univariate analysis. ORs and 95% relative confidence intervals (CIs) were calculated to determine the relevance of all potential predictors. Receiver operating characteristic (ROC) curves were used to determine the optimal cutoff values for the rate and number of central lymph node metastases (CLM) in the prediction of LLNM. According to multiple logistic regression analyses, features that were independent factors were assigned different points based on the OR to develop a scoring system, and the best point with a high sensitivity and low false-negative rate (1-Specificity) was then identified. A two-tailed *p* value < 0.05 was considered indicative of statistical significance.

## Abbreviations

LNM: lymph node metastasis
LLNM: lateral lymph node metastasis
TSH: thyroid-stimulating hormone
ROC: receiver operating characteristic
CLM: central lymph node metastases
PTC: papillary thyroid carcinoma
CND: central neck dissection
LND: lateral neck dissection
CT: computed tomography
MRI: magnetic resonance imaging
ATA: American Thyroid Association
FNAB: fine-needle aspiration biopsy
